# Engaging local health research communities to enhance long-term capacity building in Brazil

**DOI:** 10.1136/bmjgh-2021-007131

**Published:** 2021-10-20

**Authors:** Luiza Helena Madia Lourenço, Bonny Louise Baker, Antonio Gregorio Dias Junior, Nina E Jamieson, Roque Pacheco de Almeida, Ricardo Queiroz Gurgel, Cristiane Campello Bresani Salvi, Clécio Gabriel de Souza, Egmar Longo, Bruno Gomes, Mariana Rocha David, Márcio Galvão Pavan, Daniele Pereira de Castro, Mauricio Lacerda Nogueira, Trudie Lang

**Affiliations:** 1 Faculty of Medicine, Universidade de Brasília, Brasilia, Brazil; 2 Centre for Tropical Medicine and Global Health, University of Oxford, Oxford, UK; 3 School of Public Health, University of California, Berkeley, California, USA; 4 Department of Medicine, Federal University of Sergipe, São Cristovão, Brazil; 5 Aggeu Magalhães Institute, Oswaldo Cruz Foundation, Recife, Brazil; 6 Faculty of Health Sciences - Trairi, Federal University of Rio Grande do Norte, Santa Cruz, Brazil; 7 Oswaldo Cruz Institute, Oswaldo Cruz Foundation, Rio de Janeiro, Brazil; 8 Faculty of Medicine of São José do Rio Preto, São José do Rio Preto, Brazil

**Keywords:** Public Health, Arboviruses, Dengue, Yellow fever

## Abstract

The ‘2019 Research Capacity Network (REDe) workshop series’ was an initiative led by Brazil-based REDe coordinators and The Global Health Network (TGHN) in partnership with Brazilian researchers interested in arboviruses. This workshop initiative has provided crucial training to the local research community offering transferable skills to effectively respond to health emergencies, with an impact beyond arboviral diseases, as evidenced by further activities undertaken during the COVID-19 pandemic. The success of this approach resulted from several factors, especially the workshops’ local leadership and the combination of in-person training with online sharing of the resources generated in the local language. Analytics data from REDe online platform evidenced the wider reach of the shared resources to a larger audience than the workshop attendees. Importantly, the impact of this approach extends beyond the workshop series per se, with workshop participants afforded access to wider training, career development and collaborative opportunities through REDe and TGHN platforms. In addition, this initiative design resulted in the development of new collaborations between the workshop leaders and other local researchers, who have been jointly writing research projects and applying for grants. As a result, REDe has become a highly dynamic community of practice for health researchers in the region, strengthening the research culture and improving connectivity. Here, we describe the design and implementation of this initiative and demonstrate the value of integrating local expertise, and a practical workshop series format with digital dissemination of research resources and training materials to generate a vibrant and robust community of practice.

**Summary box**

- Low- and middle-income countries (LMICs) have an unmet need for research training despite strong local and regional expertise being available, lack of support is a barrier to strengthening capacity.
- International organisations interested in promoting training opportunities in LMICs must integrate local experts and important aspects such as removal of language barriers into their planning strategy.
- A blended format of in-person training combined with online knowledge dissemination is an effective way to widen the outreach of locally relevant learning resources to professionals who would otherwise have limited access to such materials.
- Supporting such interactive capacity strengthening activities is valuable to foment effective and dynamic regional networks, which are essential components of a timely response to new emerging threats.

## Introduction

Global health emergencies, such as the recent COVID-19 pandemic and Zika epidemics, produce devastating consequences, especially when combined with the diversity of social and epidemiological contexts present in low-income and middle-income countries (LMICs). These outbreaks also serve to highlight the inequality that exists in health research, in terms of where research happens and who directly benefits from it. The role of international organisations and funders in the response to such emergencies, by issuing protocols and recommendations, is vital but can be disconnected from the realities that exist within low-resourced healthcare settings. Both Zika and COVID-19 have taught us that a meaningful response requires local ownership of evidence and public policies, which in turn can only arise from a strong local research culture.^1^

Strengthening research capacity by developing and supporting ‘communities of practice’ is a strong mechanism to guide, advance and sustain a robust research environment. A community of practice is a group of people who solve a common problem by learning from each other. If a region does not have strong research skills within their healthcare system, they are unable to respond to a new disease outbreak by identifying and characterising any emergent new threat. Therefore, strengthening of research capacity must be promoted in a sustainable and constant way, as the importance of research preparedness in outbreak responses has repeatedly been shown—recently by the efforts to deal with the COVID-19 pandemic.^2^ As a result, these efforts positively and significantly impact public health, improving the local research capabilities of teams to detect and respond timely to any new emerging outbreak.^3^ Hence, the Research Capacity Network (REDe) was established in 2016 by the Global Health Network (TGHN) and the three European Union-funded Zika Consortia (ZikaAlliance, ZikaPLAN and ZikaAction) as a community of practice for researchers and wider healthcare workers across Latin America and the Caribbean. The aim of REDe is to embed research skills and experience to tackle the everyday burdens of disease in healthcare settings where the ability to collect evidence is limited.

Here, we report a capacity strengthening initiative to develop the research skills base and share expertise in tackling arboviruses, which still create overwhelming disease burdens in LMICs. Brazil-based REDe coordinators and selected workshop proponents ran a series of specialist skills workshops hosted across Brazil in 2019, focused on providing training in situ and generating an online repository of resources in Portuguese. These resources were developed by the expertise held within these participating regional teams, to the benefit of wider research communities, in-country and in other Portuguese-speaking nations. TGHN, the wider platform in which REDe is inserted, provided support to this initiative with a team based in the UK. This article describes the methodology and impact of this initiative and provides a guide for other international organisations keen to contribute in a truly sustainable manner to research capacity strengthening in LMICs.

## Local context is essential

To embed research abilities and engagement within a healthcare setting, the community and entire research team should be involved, to consider, adapt and work within the local context and local needs. One critical consideration, often overlooked by the Global North, is language. In fact, the lack of training materials provided in local languages is often undermined, and negatively impacts on the participation of healthcare professionals and members of the community in research and appropriate response to health emergencies. This is a familiar problem in Portuguese-speaking countries, which are spread in the Americas, Africa, Europe and Asia. Of note, Brazil houses approximately 73% of the global Portuguese-speaking population.^4^

Besides cultural aspects, the focus of such initiatives should be aligned with local health priorities and public health agendas. The theme of this workshop series was a prominent contemporary global health emergency in Brazil: arthropod-borne viruses (ie, arboviruses) and the diseases caused by them. Since 2014, significant outbreaks of chikungunya virus appeared in Brazil,^5^ but national data on its incidence and chronicity are still lacking.^6^ In 2015, a Zika virus epidemic infected ~1 million people, with 3000–4000 suspected cases of Zika congenital microcephaly across Latin America,^7 8^ 90% of which is in Brazil.^8 9^ Between 2016 and 2018, yellow fever cases also peaked, with 2139 confirmed human cases in the country.^10^ In 2019, there were 1.5 million dengue cases in Brazil (600% rise compared with 2018).^11^ Recurrent outbreaks of circulating arboviruses continue to significantly affect and undermine populations and health systems, especially in the absence of effective vaccines and therapies.

Finally, the identification of local partners is another important aspect to design a meaningful capacity strengthening initiative. Two REDe coordinators based in Brazil led this initiative with collaborative support from TGHN, which was responsible for general guidance on the establishment of training initiatives, online communication tools and general IT support. The local coordinators’ expertise was central to designing a mapping strategy to identify research groups across Brazil who might engage and contribute to this initiative. Where available, names and contact details for scientists acknowledged in documents from the Brazilian Ministry of Health produced during the Zika virus outbreak were gathered, as well as the named partners and collaborators within the EU-funded Zika consortia. In addition, scientific publications authored by these researchers were used to identify other leading scientists in the field using search engines such as PubMed. A list of 87 researchers working in the field of arboviruses across Brazil was assembled.

## Identifying engaged local leaders

Our aim was to identify local researchers in the field of arboviruses and support them to undertake these capacity strengthening initiatives to share their expertise, engage with international colleagues and widen their network. Therefore, a targeted promotion to launch this workshop initiative was sent to existing members of the REDe Network and the to the assembled list, inviting researchers to submit proposals (with funding awards available up to US$2000). We accepted applications in Portuguese to encourage maximum access. Collaborative proposals were encouraged, and researchers were given a 1500-word limit to describe their programme proposal. Applicants were given 2.5 weeks to submit proposals and a reminder was issued 5 days before the published deadline. Submissions were made to the central REDe email account and were scored by a panel according to pre-determined criteria (table 1). Only proposals with a minimum score of 50% across all criteria were considered. Applicants were informed about the status of their proposal within 2 weeks of the submission deadline.

**Table 1:** Scoring criteria used for evaluation of submitted applications

Criteria and indicator	Weight
Proposal	40%
Alignment with REDe’s goals and compatibility with proponent profile	
Capacity building experience	20%
Previous experience with capacity building initiatives	
Collaboration	10%
Listed collaborators should add value to the proposal goals	
Host site	10%
Necessary basic infrastructure available to host workshop	
Target audience	10%
Diversity of health professional roles	
Date	5%
Proposed date in 2019	
Research expertise	5%
Publication record in the field	

Points were allocated to the criteria below according to the relevant indicator. The predefined weighting matrix used to calculate the final score is depicted for each indicator.

**Figure 1:**
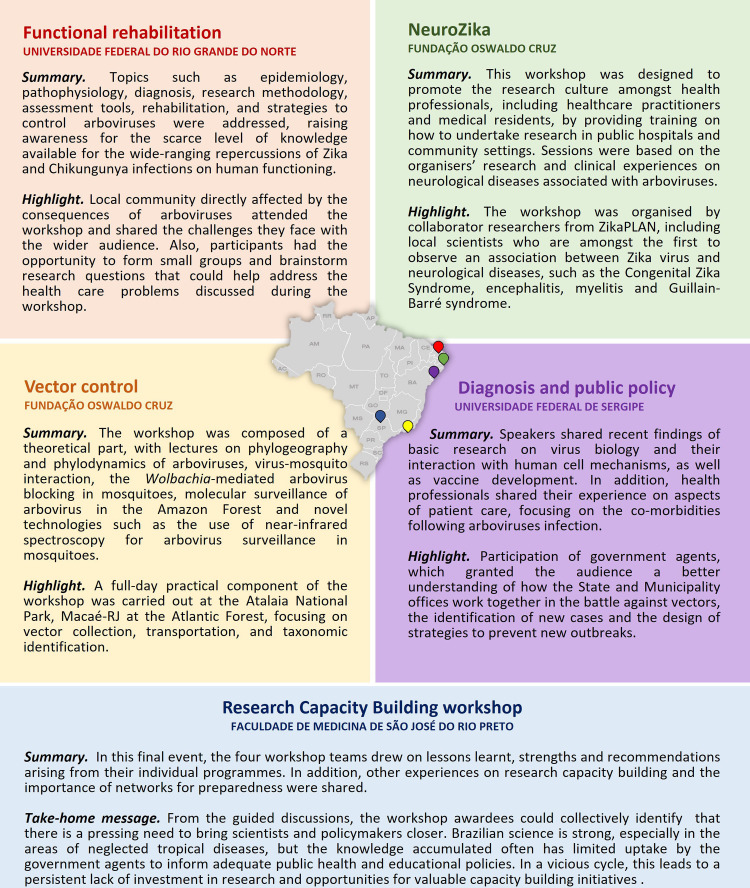
Institutional, geographical and thematic diversity of the 2019 Brazil workshop series. Brazil is the fifth largest country in the world, with important regional differences that strongly affect the consequences of diseases. In acknowledgement of this regional variation, this workshop initiative funded proposals from different geographical areas of Brazil, as shown in the map by the colour-coded pins. Five Brazilian public institutions hosted workshops with a broad range of programmes under the theme of arboviral disease, in five different states: Rio Grande do Norte (RN), Pernambuco (PE), Sergipe (SE), São Paulo (SP) and Rio de Janeiro (RJ).

With this selection process, we were able to identify research group leaders in Brazil who could design relevant programmes to enhance capacity, offering research skills training to local researchers and healthcare professionals who were not previously involved with research. Based on the final scoring, five proposals from different Brazilian institutions were successfully awarded. Each were focused on subspecific topics within the field of arboviral diseases and related skills, as described in figure 1.

## Workshop demographics

As a means to capture basic demographic information and professional characteristics of participants, registration to attend each workshop was made available as an online form hosted through the REDe platform (https://rede.tghn.org/). Consent was sought for future use of the collected data in reporting of this initiative. For the purposes of this article, data obtained from the registration forms of the four skills-based workshops were combined and data from the concluding consensus meeting was not included.

**Figure 2:**
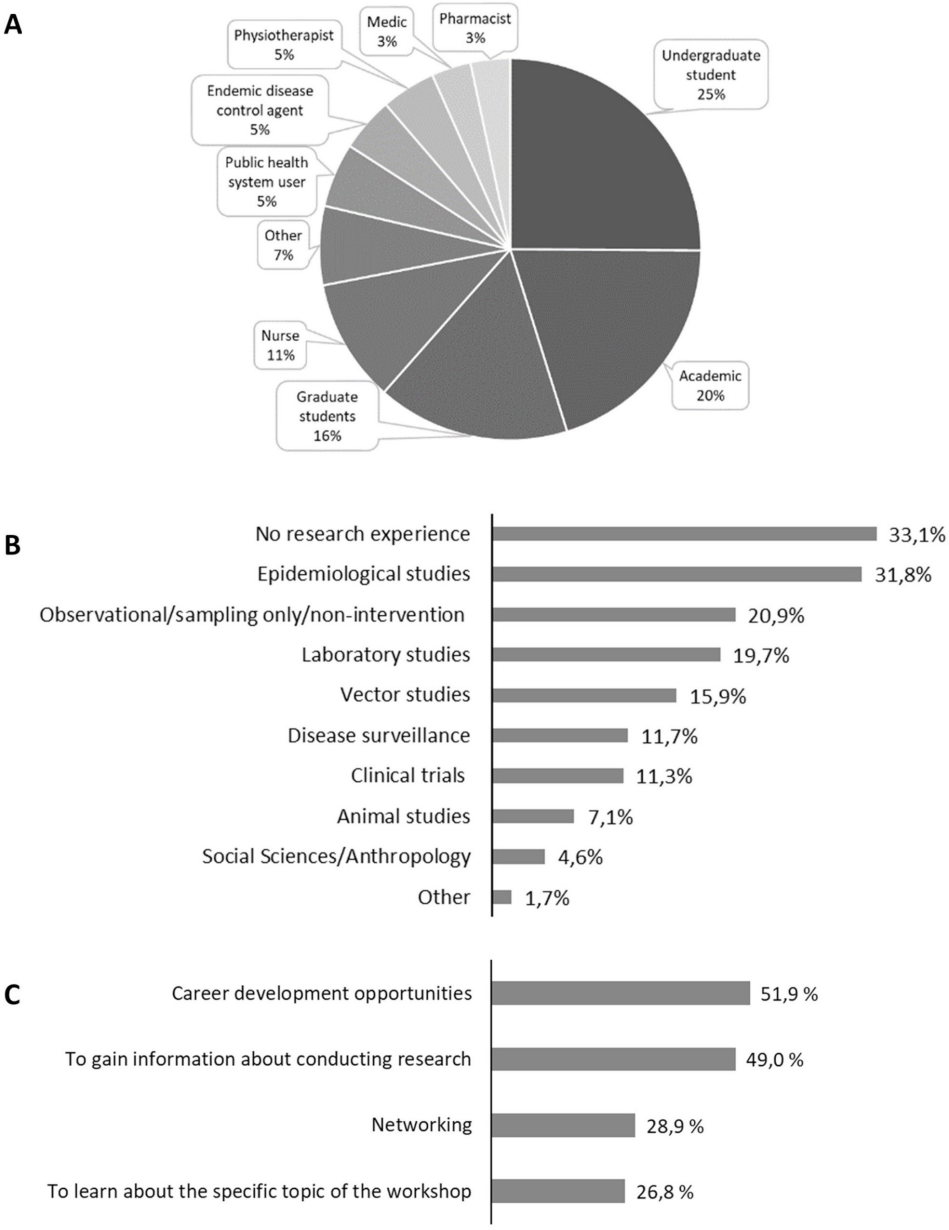
Demographics of the 239 participants registered for the four workshops. The registration requested information concerning current role (A), research experience (B) and motivation to attend (C). Percentage was calculated considering the total number of answers for each indicated option divided by total number of participants; multiple answers were accepted in questions B and C.

In total, 239 people registered across the four skill-based workshops, including professionals and students (figure 2A). From those, 163 attended the workshops (68.2% attendance rate). Within the general theme of ‘arboviruses’, participants had varied research expertise, ranging from vector studies to clinical trials (figure 2B). Notably, 33% of the participants reported having no previous research experience, which indicates the local need for such capacity strengthening efforts and confirms that this series effectively reached an important target group. In addition, the most common reasons for registering to attend were expressed as ‘career development’ and ‘research methodology training’, but interest in ‘networking’ and ‘learning about the specific scientific topic’ were also reported (figure 2C).

## Impact of the workshops

**Figure 3:**
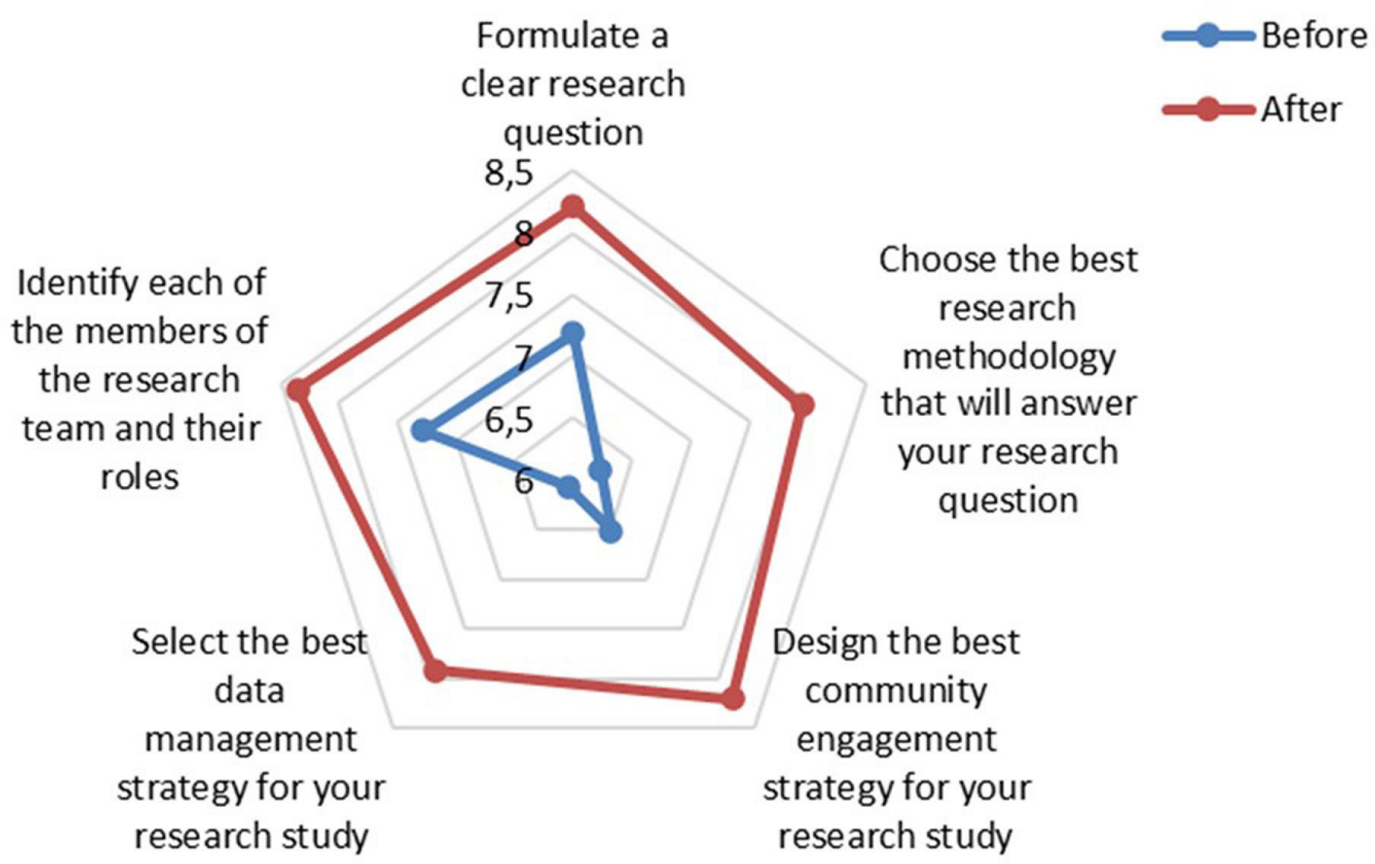
Workshops impact on research skills assessed by self-reported questionnaires. Participants were asked to evaluate their research skills in advance and after attending the workshops (n=82). The reported values correspond to the average score among the participants, who were asked to score their perceived competence using a scale from 0 (low) to 10 (high) for each question on the listed research skill.

Participants were asked to complete an optional feedback questionnaire at the end of the workshop session, and a total of 82 evaluations were submitted (50.3% response rate, from 163 attendees). A set of questions about research skills was duplicated in both the registration and feedback questionnaires, which offered a comparative indicator for the workshop impact reported by participants. When comparing the answers given by the same 82 participants on both questionnaires, there was an improvement in the self-reported grade on all research skills analysed. These included the ability to (1) formulate a clear research question, (2) choose the most appropriate methodology, (3) design community engagement strategies, (4) identify team members and their roles and (5) select the most appropriate data management system (figure 3).

Descriptive feedback was also obtained through open questions in the feedback questionnaire, which supported the quantitative data. One participant stated that ‘The talks related to fieldwork helped me a lot to re-evaluate my own graphs and maps.’ Another participant wrote that ‘The workshop will help me in the dissertation writing process as well as fostering my future research projects.’ Other participants pointed out the importance of such practical sessions, suggesting they should be longer in duration. Of note, many participants highlighted the effectiveness of learning from experts through interactive discussions.

To conclude the series, the REDe team organised the ‘Research Capacity Meeting’ review and consensus event. The aim was to provide a forum where the workshop awardees could collectively identify the main challenges and hurdles for research capacity strengthening in Brazil and discuss ways to overcome them. This meeting was hosted prior to the international conference ‘Emerging infections in the Americas—common interests and collaboration between Brazil and USA’, held in a partnership between the Faculty of Medicine of São José do Rio Preto and the University of Texas Medical Branch. REDe provided additional funding for the workshop leaders to attend this meeting, which was an opportunity for international networking. During presentation sessions and discussions, the workshop teams drew on lessons learnt, strengths and recommendations arising from their programmes. In addition, as part of this session, other experiences on research capacity strengthening and the importance of networks for preparedness were shared by representatives from the International Severe Acute Respiratory and Emerging Infection Consortium, the Microcephaly Epidemic Research Group and the Sustainable Sciences Institute. From the guided discussions, it was clear that there is a pressing need to bring scientists and policy-makers closer, creating a constructive interface for open dialogue. The overall message was that Brazilian science is strong, especially in neglected tropical diseases, but the knowledge accumulated often has limited uptake by the government to inform adequate public health and educational policies. In a vicious cycle, this leads to a persistent lack of investment in research and opportunities for capacity strengthening initiatives at institutional and national level.

## Sustaining the support

The design of this initiative afforded an opportunity to generate strategies for promoting research capacity strengthening locally, as well as recognising and improving the visibility of local expertise, empowering regional researchers. This was successfully achieved through the open and free sharing of training materials generated throughout the workshops, and subsequently hosted through the REDe online platform. To improve visibility for the institutions and individual groups, dedicated areas for each workshop were created to host photos, presentation slides from the talks, summary reports and testimonial videos from participants. Other outputs were also generated, including a short documentary about the management of Guillain-Barré syndrome during the recent Zika epidemic in Brazil. This platform has provided the mechanism for researchers and teams to showcase their expertise with researchers and health organisations globally, and thereby strengthening this community of practice.

**Figure 4:**
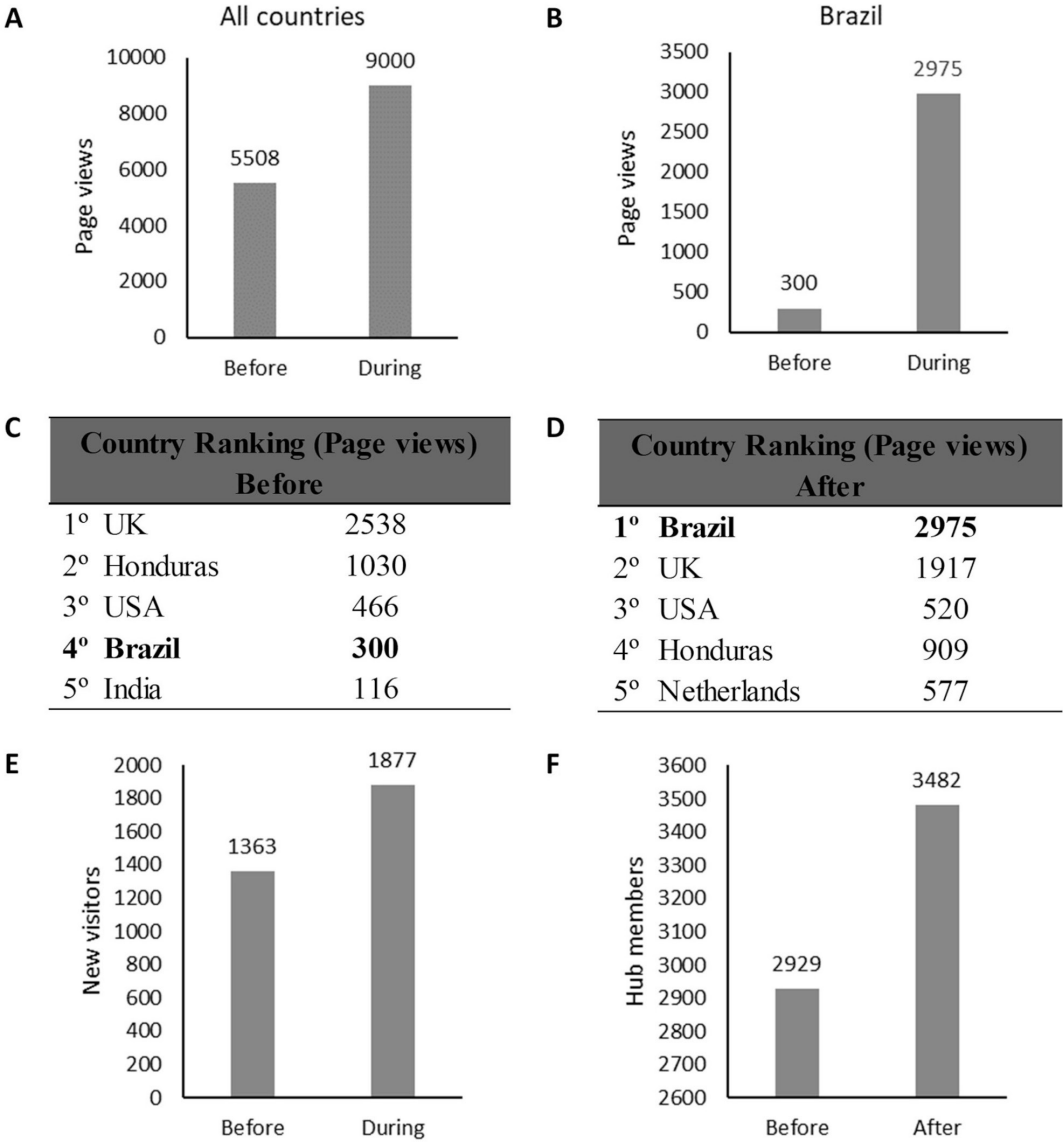
The impact of the 2019 workshop series for promoting access to the digital REDe hub. Data provided by analytics from across the 4-month period of actively hosting workshops (September–December 2019) was compared with the 4-month period before the workshops (May–August 2019) and parameters such as total page views (A), page views from Brazil (B), country ranking by page views–before (C) and after (D), new visitors (E) and new members (F) were assessed. REDe, Research Capacity Network.

Analytics data generated using a tracking code, indicates that wide knowledge-transfer was achieved through this initiative, initially indicated by a rise in traffic and access to the REDe hub during the workshop series (figure 4). The comparison was made between the 4-month period in the run up to the workshops (May–August 2019) and the 4-month period which included the hosting time frame for the workshop series (September–December 2019). Total page views on the Hub increased by 38.8% during the active workshop hosting. Data specifically from Brazil showed that page views from this country rose from 300 to 2975, ranking as the top country for page views when compared with the rest of the world. Meanwhile, analysing the data on users accessing the page for the first time (using a different device/browser also generates a new user ID) irrespectively of the country of origin, the number of new visitors increased by 37.7% (from 1363 to 1877). Importantly, this resulted in an increase in the number of REDe members, that is, users who have registered for an account in the platform, from 2929 to 3482 (553 new members).

Our evidence suggests that the process of capacity strengthening can be supported and expanded through a model of classroom and practical workshops integrated with a digital component that provides ongoing support and access to tools and further training. However, it is an important finding that the number of new visitors to the platform was substantially higher than the number of workshop attendees, indicating that those who were not able to attend the workshops benefited from the opportunity to access the online content. Therefore, this digital component helped meet a demand, by mitigating issues with accessibility and the limitations presented by on-site training, such as travel expenses or time commitments. This is especially important in times of global health emergencies as shown throughout the COVID-19 pandemic, as gatherings are limited, and in-person training opportunities heavily restricted.

Additionally, increased access to the REDe hub by LMIC researchers indicates the value of this approach in serving to meet a pressing and unmet training need. The digital platform also offers and signposts to further free skills-based eLearning, access to free webinars and open virtual workshops, networking opportunities across diverse disciplines and settings, and support with career development. Critically, this forum also assists with the dissemination of funding calls and acts as an effective forum for fostering international collaborations.

In addition, the concluding consensus meeting created a unique opportunity for the workshop leaders to establish a local working group among themselves. Together with the REDe team, the group generated an integrated project proposal, encompassing the specific research priorities from within their individual fields. The project also includes a capacity strengthening component focused on active communication mechanisms necessary to foster science-based policy-making and public understanding of the importance of science. Now, this strong and integrated proposal can be sent to international funding calls and potentially secure funding, which represents a pragmatic benefit at a time when Brazilian researchers meet with a decrease in public funding for research, and securing funding to support research efforts through international grants has increased emphasis and dependenace.^12^

The value of strengthening communities of practice such as REDe is further accentuated by the role it has played as the Latin America regional lead for TGHN COVID-19 Research Implementation Hub (https://coronavirus.tghn.org/). As a result, a working group formed by researchers from Portuguese-speaking countries was established, creating critical dialogue and generating position papers to highlight their countries’ response to COVID-19.^13^ Furthermore, a second series of REDe workshops replicating the approach described above launched across 2020–2021. One of the sessions in this subsequent series is providing training for healthcare professionals running a COVID-19 clinical study. Contributing directly to the work underpinning the pandemic response, teams for this workshop series feature existing partners as well as new collaborators, indicating that this community is growing in the face of persistent new challenges. This demonstrates the value of building a community of practice in Brazil through REDe that is active and offers agility to address novel research challenges whenever they arise.

## Lessons learnt

The 2019 REDe workshop initiative serves as a ‘proof of concept’ of a mechanism to enable lasting research capacity in challenging settings. From this experience, we have learnt that combining in-person training with further online knowledge dissemination is key to widen the outreach of learning resources. This is the case especially in countries in which English is not widely spoken: it is paramount to support local experts to produce relevant training resources in local languages, generating accessible materials to teams who would otherwise have limited access to high quality educational content. Furthermore, hosting and dissemination of such resources in an online platform is effective to attract more members to a growing community of practice. In the long term, these mechanisms are essential to improve health indicators as they bring cutting-edge scientific findings to healthcare professionals, health systems and ultimately to patients. A further important merit to the approach described was the organic development of a working group among the workshop leaders, showing such initiatives are important to foster collaboration among local experts by bringing researchers closer. Collectively, these factors have contributed to the development of strong and active research awareness and engagement, as well as strengthened research skill bases. All of this positively affects research even further than the initial focus and help addressing new and emerging health threats. We could observe this effect in the response of the REDe community to COVID-19 through the TGHN COVID-19 Research Implementation Hub, a network of researchers that emerged from REDe.

## Conclusion

Our experience shows that LMICs such as Brazil still have a pressing and unmet need for research training, with incredibly strong local and regional expertise apt to share and deliver these learning experiences. However, effective support that is truly sustainable and transformative to research capacity strengthening in LMICs remains scarce.^14^ This includes the lack of funding to produce training resources in local languages and on relevant subjects, as well as the availability of platforms to widely disseminate them and provide an environment for research collaboration. Mechanisms to support the sharing of such excellence like the approach described above can catalyse effective and dynamic regional networks, with vital transference across disease areas and organisations, creating better capabilities to fight enduring but also new emerging threats.

## Data Availability

Data sharing not applicable as no datasets generated and/or analysed for this study.
